# Hemiballism-hemichorea induced by ketotic hyperglycemia: case report with PET study and review of the literature

**DOI:** 10.1186/2047-9158-3-14

**Published:** 2014-07-10

**Authors:** Yuyan Tan, Xiaoyu Xin, Qin Xiao, Shengdi Chen, Li Cao, Huidong Tang

**Affiliations:** 1 Department of Neurology, Ruijin Hospital Affiliated to Shanghai Jiao Tong University School of Medicine, Shanghai 200025, China

**Keywords:** Hemiballism-hemichorea, Ketotic hyperglycemia, Primary diabetes mellitus

## Abstract

Hemiballism-hemichorea (HB-HC) is commonly used to describe the basal ganglion dysfunction in non-ketotic hyperglycemic elderly patients. Here we report two elderly female patients with acute onset of involuntary movements induced by hyperglycemia with positive urine ketones. We described the computed tomography and magnetic resonance imaging findings in these two patients, which is similar to that of non-ketotic hyperglycemic HB-HC patients. FDG-PET was performed and the glucose metabolism in the corresponding lesion in these two patients was contradictory with each other. We tried to clarify the underlying mechanisms of HB-HC and explain the contradictory neuroradiological findings in FDG-PET as being performed at different clinical stages.

## Introduction

Ballism and chorea can result from a varity of conditions, including cerebrovascular, metabolic, neurodegenerative, infectious, toxic, immunologic disorders, as well as non-ketotic hyperglycemia [1-3]. Hemiballism-hemichorea (HB-HC) is commonly used to describe the basal ganglion dysfunction in non-ketotic hyperglycemic elderly patients. It is an unusual clinical entity characterized by continuous, proximal and distal, involuntary movement, particular neuroradiological findings in brain computed tomography (CT) and magnetic resonance imaging (MRI) [3-6]. Usually HB-HC is induced by non-ketotic hyperglycemia [1-4,7-9], here we report two cases of Chinese elderly women who manifested as HB-HC induced by hyperglycemia with positive urine ketones. We also described CT, MRI and FDG-PET findings in these two patients. They had the particular hyperdensity in the striate area contralateral to the side of HB-HC in CT, and high signal intensity in the putamen in MRI T1-weighted (case 2). The FDG-PET findings in these two patients were contradictory, one patient had decreased glucose metabolism in the lesion while the other patient had hypermetabolism. The mechanism of this syndrome is still not clear. Possible explanations for the pathophysiology and the contradictory radiological findings are also presented.

### Clinical cases

#### Case 1

**Figure 1:**
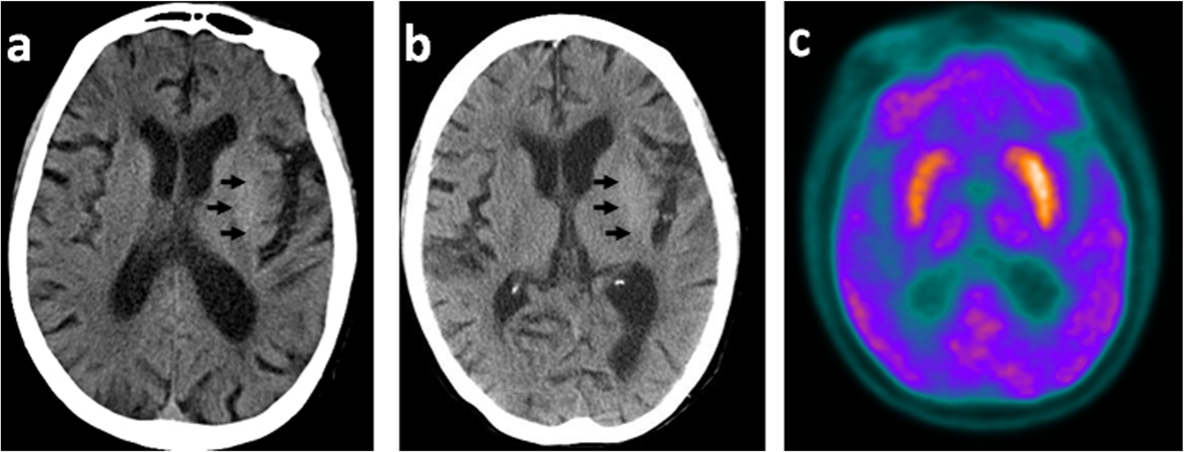
**Images from patient 1. a.** Brain CT (axial) the same day of onset, showing a hyperdense lesion in the left putamen (arrow); **b.** Brain CT(axial) 4 days after onset, revealing a slight high-density in the same area, but the density decreased compared with the initial CT (arrow); **c.** The FDG-PET scan (axial) 9 days after onset, revealing hyper-perfusion in the left side of the basal ganglion.

Our first patient was an 83-year-old woman who had had poorly controlled diabetes for more than 10 years. On 05 November 2011, she had an acute onset of right upper limb involuntary movements after a fall without head injury. There was no loss of consciousness, disorientation, confusion or urinary and fecal incontinence. She was presented to the emergency room of another hospital. On admission she was found to have a blood glucose level of 16.4 mmol/l. Her urine ketones were positive (++++) with normal arterial blood gas analysis. All other laboratory tests were normal. Initial brain CT showed a hyperdense lesion in the left putamen (Figure 1a). The patient was treated with insulin and haloperidol and the symptom was slightly relieved. We saw her 4 days later. Neurological examination showed that patient had involuntary movements, continuous flexion–extension and rotational movements, no evidence of weakness or sensory disturbance. Deep tendon reflexes were normal and plantar responses were negative. The movements could not be suppressed voluntarily but disappeared during sleep. Her fasting blood glucose concentration was 10 mmol/l with glycosylated hemoglobin A1c (HBA1C) of 12.6% at that time. Urine ketones were negative. Brain CT was performed again (4 days from onset), the high-density of the lesion decreased compared with the initial CT (Figure 1b). After written informed consent was obtained from patients, FDG-PET was performed 9 days after onset. Regional cerebral glucose metabolism was significant increased on the left side of the basal ganglion (Figure 1c). Through control of blood glucose and haloperidol, the symptoms were relieved and the patient was discharged 11 days later.

#### Case 2

**Figure 2:**
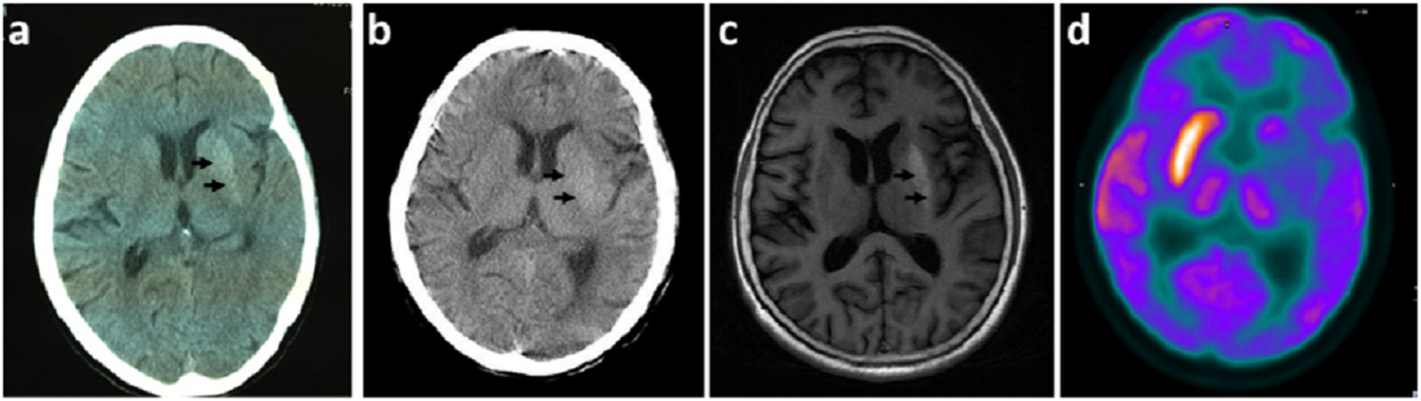
**Images from patient 2. a.** Brain CT (axial) the same day of onset, showing high density in the left putamen (arrow); **b.** Brain CT (axial) 24 days after onset, showing slight high-density in the same area (arrow), but the density decreased compared with the initial CT; **c.** MRI (axial) of the head 62 days after onset, demonstrating hyperintensity on T1-weighted images in the putamen (arrow); **d.** The FDG-PET scan (axial) 55 days after onset, revealing hypo-perfusion in the left side of the basal ganglion.

The second patient was a 59-year woman with poorly controlled diabetes for 10 years. On 10 October 2012, she had an acute onset of continuous, involuntary movement of the right side of the body. The involuntary movements were non-suppressible and increased with emotional tension and ceased after sleep. At that time, fasting blood glucose was 20 mmol/l. Her urine ketones were positive (++++) with normal arterial blood gas analysis. Brain CT showed a hyperdense lesion in the left basal ganglion region (Figure 2a), which was misdiagnosed as hemorrhage at another hospital. The blood glucose was controlled in the range of 10-15 mmol/l. The symptoms were not relieved through administration of haloperidol and Tiapride. On 23 November 2012, she was admitted to our hospital. Neurological examination showed right-sided ballism-chorea movements without abnormal pyramidal or sensory signs. Her serum glucose concentration was 15 mmol/l with an HBA1C of 13.2%. The urine ketones was positive (+) on 24 November. The arterial blood gas analysis was normal. The blood glucose was well controlled by insulin and the urine ketones became negative on 28 November, but the symptoms were not relieved. CT scan (24 days from onset) showed slightly high attenuation of the left putamen, and the density was decreased compared with the initial CT scan (Figure 2b). MRI of the brain (62 days from onset) showed a strong T1 -weighted hyperintensity in the left lenticular nucleus, mainly in the putamen (Figure 2c). After written informed consent was obtained from patients, brain FDG-PET was performed 55 days after onset, regional cerebral glucose metabolism was significantly decreased on the left side of the basal ganglion (Figure 2d). The blood glucose was controlled by insulin. Artane, Clonazepam and Baclofen were also administered to relieve the involuntary movement. The symptoms were moderately relieved at 2 months after onset.

## Discussion

HB-HC has been described mostly in Asian elderly women patients induced by non-ketotic hyperglecemia [6,8-11]. Here we report two Chinese elderly female HB-HC patients induced by hyperglycemia with positive urine ketones and normal arterial blood gas analysis. Ketone body includes acetoacetate, β-hydroxy butyric acid and acetone. Urine ketones mainly detect acetoacetate, while blood ketones mainly detect β-hydroxy butyric acid. Although we didn’t detect the blood ketones, the positive urine ketones partially indicated that ketones metabolism was interrupted in these two patients. To the best of our knowledge, only one paper from Dilek Ersil Soysal et al reported an old-age female patient with transient monoballismus during an episode of hyperglycemia with positive urine ketones [12].

The underlying mechanism for hyperglycemia associated HB-HC is unknown. Possible mechanisms include cerebral vascular insufficiency [8], hyperglycemic or hyperosmolar insult [13], microbleeding [14], interruption of GABA transmission [15-17], autoimmune-mediated inflammatory process [18], etc. A vascular event is regarded as a possible cause of the striatal lesions based on the sudden onset [19,20] and the particular neuroradiological findings, high density in striate area in CT and high signal intensity in putamen and/or caudate in T1 weighted MRI. However, demyelination, calcium or other unknown metabolites accumulation have also been regarded as possible causes for the radiological features [21-23].

We used CT and FDG-PET to examine these two patients and MRI for case 2. Both patients showed hyperdensity which is well below that for hemorrhage in the putamen contralateral to the affected side on CT scan. MRI finding in case 2 showed high signal intensity in the putamen contralateral to the side of HB-HC in T1-weighted images. The FDG-PET findings in these two patients were different. In case 1, FDG-PET was performed at 9 days after clinical onset, the involuntary movements was slightly relieved compared with the first day of admission. The rates of cererbral glucose metabolism were significantly increased on the corresponding side. In case 2, the FDG-PET study was performed on 55 days after onset and the symptoms was not well controlled. PET study showed that regional cerebral glucose metabolism was markedly decreased in the corresponding side of the basal ganglion. In Jung Lung Hsu’s report [8], FDG-PET was performed in three hyperglycemia induced HB-HC patients at 3 weeks, 5 weeks, and 7 months after clinical onset. The rates of cerebral glucose metabolism were markedly reduced in the corresponding lesions. This change provides direct evidence that cerebral glucose metabolism in the lesion site is decreased, which supports the notion of cerebral glucose metabolic failure in the lesions. However, contralateral striatal hypermetabolism was found in FDG-PET scan of chorea caused by other conditions, like primary antiphospholipid syndrome, Contraceptives [24]. Thus, which condition reflects the true pathophysiological metabolic changes in hyperglycemia-induced HB-HC? SPECT studies provided some clues. Seung-Hun Oh et al [6] reported the results of brain SPECT studies showing cerebral blood perfusion in chorea patients associated with non-ketotic hyperglycemia. Four of them showed hypoperfusion of the basal ganglia on the contralateral side (range = 8 days–4 months), in other four patients, hyperperfusion found in the initial study (range = 6–20 days) had changed to hypoperfusion on the follow-up study. The hyperperfusion of the basal ganglia observed in the earlier clinical course can be explained by increased cerebral blood flow because of vascular autoregulation, the hypoperfusion of the basal ganglia at the later clinical course may be caused by a neuronal metabolic derangement due to hyperglycemia, vascular insufficiency, or both. So in our first patient, PET was performed in comparatively earlier clinical stage (9 days from onset), the rates of cerebral glucose metabolism was increased might due to the increased blood flow and high blood glucose, which is in accordance with the SPECT study. In case 2 and Jung Lung Hsu’s report [8], PET were performed at the later clinical stage (range > 3 weeks), the decreased cerebral glucose metabolism may be caused by the irreversible damage by metabolic derangement and ischemia of vascular insufficiency. Although we lack the follow-up PET study, we think that whether hyperglycemia is well controlled or not contributes to the changes of metabolism in PET. When the hyperglycemia is well treated, the chorea usually disappears within a few days and might prevent the irreversible damage to the neurons.

The theory of interruption of GABA transmission might provide the molecular mechanism for the involuntary movement. In non-ketotic hyperglycemia, the shift to anaerobic metabolism causes brain to utilize amino butyric acid (GABA) as an alternate energy substrate, which caused the rapid depletion of GABA and ultimately interrupted the GABAnergic transmission [15-17]. GABA is the neurotransmitter responsible for the inhibitory pathway especially in the indirect and direct pathway in basal ganglion. In the indirect pathway, the interruption of GABAnergic transmission from the striatum to the external segments of the globus pallidus (GPe) would cause abnormally increased GPe neuron inhibitory activity on the subthalamic nucleus (STN) [15,25]. Increased inhibition on STN would decrease its excitatory action on the internal segments of the globus pallidus (GPi), which would lead to decreased GPi neuron inhibitory action on thalamus. The decreased inhibition on thalamus would lead to increased excitatory action on cortex. Besides excitatory STN inputs [15,26], the GPi neurons also receive inhibitory afferent inputs directly from the striatum in the direct pathway. The imbalance between the indirect excitatory and direct inhibitory pathways ultimately leads to a disinhibition of the motor thalamus and caused the motor cortex over excited [3,15,16]. However, patients with positive urine ketones had disrupted ketones metabolism and higher acetoacetate level. As we know, GABA can be synthesized from acetoacetate, so it can not be depleted easily in patients with positive urine ketones. So there might be other mechanisms in the pathophysiology of ketotic hyperglycemia. Another theory first put forward by Carla Battisti et al [9] in 2009 was that hyperglycemia may directly induce alterations in dopaminergic activity (upregulation of dopamine receptors, decreased DA catabolism) in the striata of predisposed patients and dysregulation of direct and indirect pathways, which ultimately increased the excitatory effect of thalamus on cortex. This theory has been suggested by several studies in animal models [17-29]. HB-HC occurs mostly in elderly female patients indicating female is a predisposing factor. It might be related to postmenopausal oestrogen-induced alterations of GABA or dopamine receptors [3,30]. For the second patient, the HB-HC symptoms were still present although moderately relieved even after 2 months from onset, which indicated the existence of a mechanism that has long-term effect. Thus, the upregulation of dompamine receptors might be a better explanation to the second patient. Above all, we think both the interruption of GABA transmission, upregulation dopamine receptors and decreased DA catabolism are involved in the pathophysiological mechanism of ketotic hyperglycemia induced HB-HC.

Moreover, in case 1, there was another predisposing factor for the acute onset. On 05 November 2011, the patient had a sudden fall, with hipps and right hand touched the ground. There was no injury of the head and right upper limb. When she stood up by herself, the involuntatory movement started. As these two events were so close, we excluded psychogenic dystonia and posttrauma induced dystonia in this patient. The patient had poor controlled diabtes for more than 10 years, which caused damage to the blood vessel for a long time. The sudden fall became a strong stress to the already weak blood vessel. Moreover, the sudden fall can also be a stress causing sharp elevated blood glucose.

## Conclusion

We presented two elderly female HB-HC patients caused by hyperglycemia with positive urine ketones. We conclude that a combination of cerebral vascular insufficiency and metabolic derangements leading to interruption of GABA transmission, upregulation dopamine receptors and decreased DA catabolism, ultimately causing unilateral basal ganglion dysfunction. Although we lack the serial follow-up of PET studies with correlation to the changes of clinical symptoms in these patients, our PET studies combining with the previous PET and SPECT studies provided evidence for the subsequent regional metabolic failure. Ideally, serial follow-up of the PET/SPECT studies in these patients could provide further insights into the actual mechanisms underlying HB-HC.

## Competing interest

The authors declare that they have no competing interests.

## Authors’ contributions

YT participated in the diagnosis, collecting clinical information and drafted the manuscript. XX participated in the diagnosis and collecting clinical information. QX participated in the diagnosis. SC participated in the diagnosis. LC provided the patient and corrected the manuscript. HT participated in the diagnosis, collecting clinical information and corrected the manuscript. All authors read and approved the final manuscript.
